# Animal Study to Evaluate the Effect of Carbon Dioxide Insufflation on Recurrent Laryngeal Nerve Function in Transoral Endoscopic Thyroidectomy

**DOI:** 10.1038/s41598-019-45779-8

**Published:** 2019-06-27

**Authors:** Daqi Zhang, Shijie Li, Gianlorenzo Dionigi, Jiao Zhang, Chunbo Niu, Tie Wang, Nan Liang, Hui Sun

**Affiliations:** 1 Division of thyroid Surgery, China-Japan Union Hospital Of Jilin University, Jilin Provincial Key Laboratory Of Surgical Translational Medicine, Jilin Provincial Precision Medicine Laboratory of Molecular Biology and Translational Medicine on Differentiated Thyroid Carcinoma, 126 Xiantai Blvd, Changchun city, Jilin province P.R. China; 2 Division for Endocrine and Minimally Invasive Surgery, Department of Human Pathology in Adulthood and Childhood “G. Barresi”, University Hospital G. Martino, University of Messina, Via C. Valeria 1, 98125 Messina, Italy; 3 Division of Pathology, China-Japan Union Hospital Of Jilin University, 126 Xiantai Blvd, Changchun city, Jilin province P.R. China

**Keywords:** Surgical oncology, Thyroid cancer

## Abstract

Data with regard to potential recurrent laryngeal nerve (RLN) compromise caused by intra-neck CO_2_ insufflation during transoral endoscopic thyroidectomy vestibular approach (TOETVA) are missing. RLN electromyographic (EMG) profiles, metabolic and hemodynamic parameters (oxygen saturation, heart rate, blood pressure, experimental time, CO_2_ partial pressure, pH, O_2_ partial pressure), central venous pressure (CVP), airpocket temperature and pressure were recorded in a TOETVA animal model. Twelve pigs were randomly divided into different groups according to increasing CO_2_ insufflation pressures. Nerves segments were then collected for histopathology. Significant variation of metabolic and hemodynamic parameters were registered when CO_2_ insufflation pressures increased x3 and x5 the baseline parameters. Combined EMG amplitude drop and latency increase also were documented. There was no significant change in the intraluminal temperature. RLNs structure were preserved with normal axons, no fibrosis, and no vacuolization and without loss of myelinated fibers during the experiment. RLN EMG profiles (but not histology) were altered when CO_2_ insufflation pressures increased.

## Introduction

Transoral endoscopic thyroidectomy vestibular approach (TOETVA) is a natural orifice transluminal endoscopic surgery and is now being widely applied^1–3^. The advantages of TOETVA consist of improved cosmetic outcomes, median approach, near access to thyroid gland, limited tissue dissection and combined level VI and VII lymph node clearance^2,3^.

Carbon dioxide (CO_2_) insufflation is required at 6 mmHg to produce and maintain an optical working cavity that enables the dissection to proceed and lessen external mechanical retraction^4^.

Data with regard to potential recurrent laryngeal nerve (RLN) compromise caused by intra-neck CO_2_ insufflation are missing. The hypothesis of the present study is that CO_2_ may irritate the laryngeal nerves and affect the physiology of the surrounding tissue. For instance, post-endoscopic shoulder pain frequently follows a laparoscopic cholecystectomy^5^. A proposed mechanism is the transient injury of the phrenic nerve (PN) by CO_2_ during peritoneal insufflation^6^. The incidence of such pain varies from 35–50%, and in some cases, the pain has been reported to last longer than 72 hours^5–7^. The actual cause of PN irritation is the result of cellular death caused by the combination of a temperature change from the gas at 70 °F and the drying effect of the gas at 0.0002%^5–7^.

The purposes of this prospective experimental study were: (a) establish TOETVA animal model; (b) assess potential adverse effects of CO_2_ insufflation on RLN function.

## Materials and Methods

### Setting, regulations, policies and principles

The Institutional Animal Care and Use Program Committee in Research of Jilin University approved this prospective randomized experimental study, which was conducted in 2018 in accordance with the Guidelines of animal research^8^. The study was supported by the National Nature Science Foundation of China (no. 81702651), China Postdoctoral Science Foundation (no. 2017M611313), Department of Science and Technology of Jilin Province (no. 20170520018JH and 20190201225JC) and Department of Finance of Jilin Province (no. SCZSY201714 and SCZSY201504). The conduct of experimentation on living animals was exclusively under the supervision of qualified, experienced attending veterinarian personnel^8^.

### Design

Prospective randomized experimental study.

### Personnel training

Personnel involved in the present study were provided with defined experimental, thyroidal, TOETVA and neural monitoring (IONM) procedures, with adequacy of experience of more than 1.000 human and 100 animal procedures^9,10^. They were trained in standardized IONM and TOETVA technique, unusual endoscopic conditions, trouble shooting algorithms, appropriate use of endoscopy^9,10^.

### Animals breads

Twelve male species of Duroc–Landrace piglets provided by the Animal Research Laboratory Center of Jilin University, were tested.

### Sedation, analgesia, and anesthesia

Intraoperatively, animals were in supine position with the neck slightly extended. Piglets induction anaesthesia comprised 0.5 mg atropine sulphate via subcutaneous injection, and intramuscular administration of 40 mg (2 mg/kg) each of tiletamine/zolazepam and xylazine hydrochloride. Electromiographic (EMG) electrode surface endotracheal tubes (standard reinforced 7.0# internal diameter ID, Medtronic, Jacksonville, FL, USA) were properly inserted^11^. The depth and angle of contact between the endotracheal tube (ET) electrode surface and the mucosa of the vocal cord were confirmed by video laryngoscopy. Muscle relaxants were avoided during all procedures^11^. General anesthesia was maintained by using Isoflurane (2.0–3.0%) and oxygen (2.0 L/min)^11^. Animals underwent electrocardiographic monitoring. Animals were ventilated to stabilize PaCO_2_ at 35–45 mmHg. Then, ventilation was maintained at a constant level for the remainder of the experiment. Intravenous isotonic sodium chloride solution was administered at a constant rate (0.9% sodium chloride, 75 mL per hour).

### Intraoperative neural monitoring equipment

Nerve Monitoring System 3.0 (Medtronic, Jacksonville, FL, USA) software was used to record the electromyogram. Nerves were stimulated with a single-use, incrementing stimulating probe (no. 8225490; Medtronic), with an impulse duration of 100 ms and frequency of 4 Hz^11^. Real-time EMG data were obtained via continuous VN stimulation using a 2.0 mm Automatic Periodic Stimulation (APS) electrode (Medtronic, Jacksonville, FL, USA)^11,12^. The amplitude and latency waveforms were displayed separately, and the upper limit threshold for the latency (+10 percent) and lower limit threshold for amplitude (−50 percent) were depicted as separate alarm lines^12^.

### Experimental Set-up, operation, evaluations and endpoints

#### APS and central venous catheter (CVC) insertion

**Figure 1:**
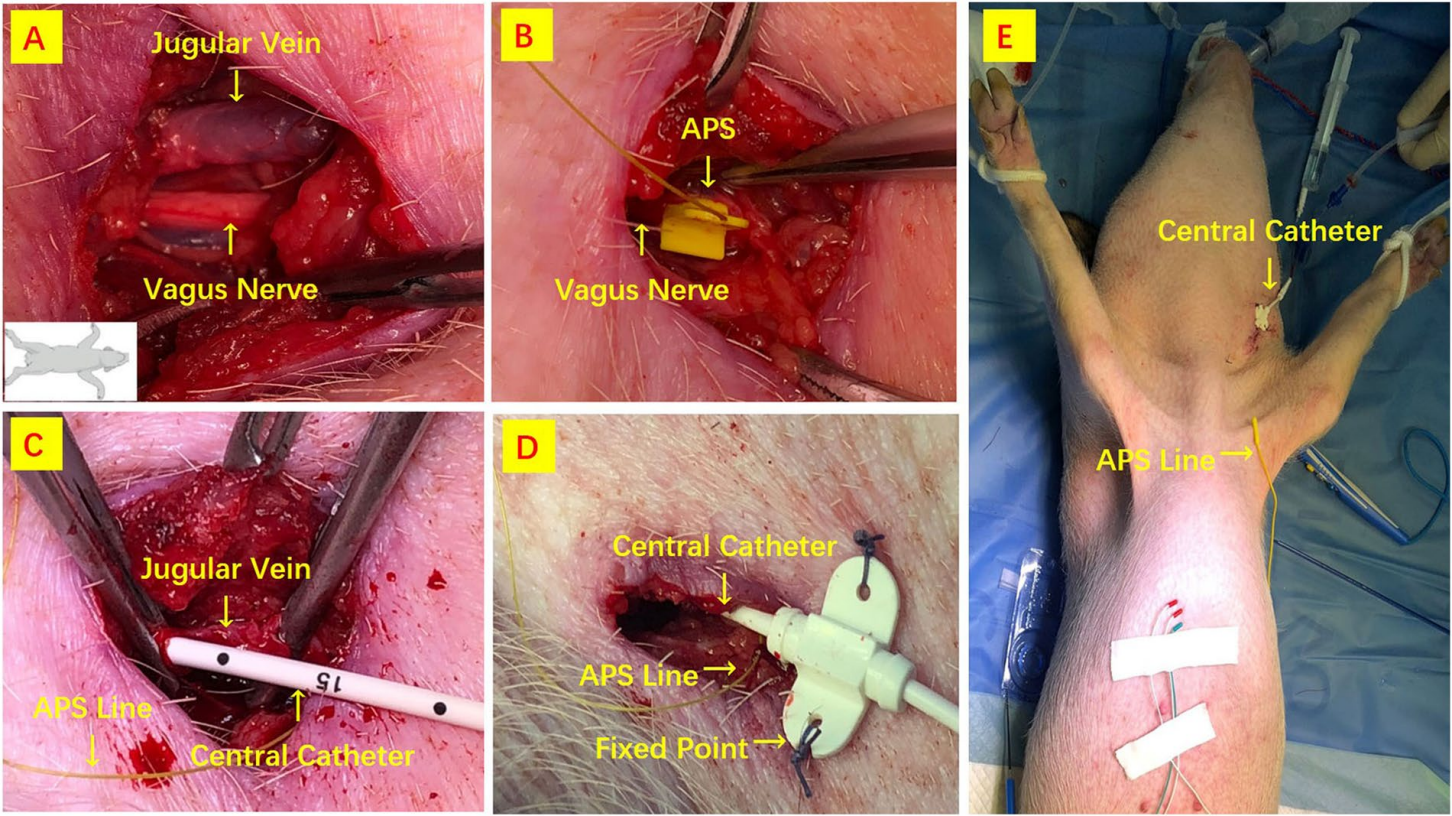
APS and CVC insertion. (**A**) A longitudinal incision is outlined above the clavicle. (**B**) APS probe is carefully placed on the VN. (**C**,**D**) CVC is tunnelled into the internal jugular vein. (**E**) CVC is fix on the the skin and connect to 0.9% saline infusion.

The APS was located outside the surgical field. Figure 1 depict APS and CVC insertion. The location of the carotid sheet was identified by landmarks and with the use of a ultrasound (US) device. A longitudinal incision of about 2 cm was outlined 5 cm above the left clavicle. VN and internal jugular vein were surgically exposed (Fig. 1A). APS probe was carefully placed on the VN (Fig. 1B). The functional integrity of the VN was confirmed by proximal and distal stimulation of APS location to verify whether the dissection or electrode placement determined VN injury (Fig. 1b). After connecting the APS electrode with the monitor system, baselines for the latency and amplitude of the evoked response were calibrated automatically to serve as control data. Stimulation frequency for C-IONM was set for every second, thus evaluating the RLN and VN constantly. During the experiment, stimulated EMG signals were registered continuously. CVC was tunnelled with disposable sterile pediatric catheter (Baihe Medical ABLE^®^, Guangdong, China) into the internal jugular vein for 10 cm and placed into superior vena cava (Fig. 1C,D). US was performed afterwards to confirm that the line was positioned inside the superior vena cava. CVC was fix on the the skin and connect to 0.9% saline infusion (Fig. 1D,E). The distal port was attached to manometer scale (Medifix^®^, B. Braun Melsungen, Hessen, Germany) for measurement of the central venous pressure (CVP). A supplementary video describes the procedure (Video 1). Baseline CVP was continuously measured in the midaxillary left line.

#### TOETVA and RLN exposure

**Figure 2:**
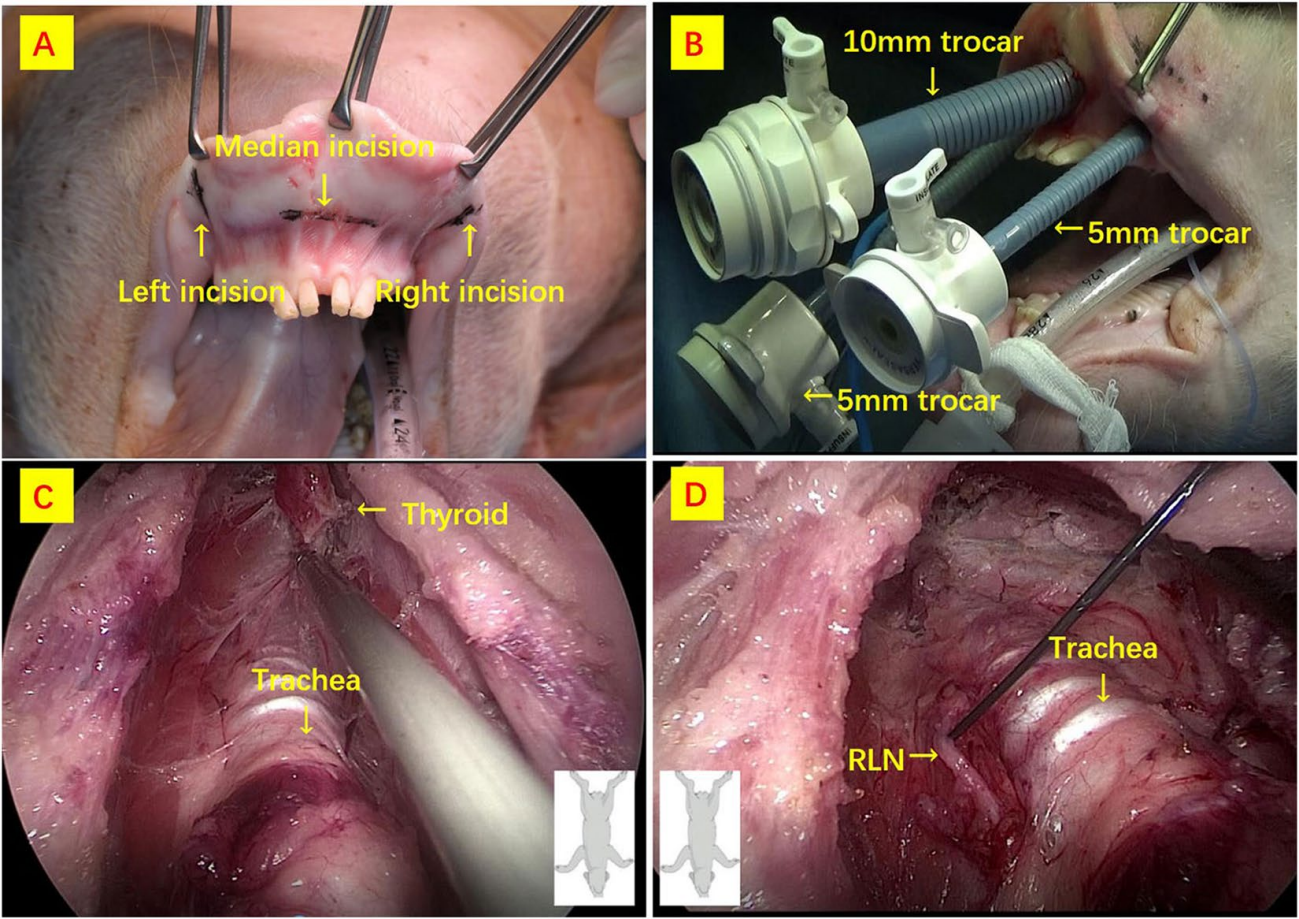
TOETVA procedure. (**A**) Surgery is initiated with a 10 mm incision in the center of the oral vestibule just above the inferior labial frenulum for the 10 mm trocar insertion. (**B**) Two 5 mm trocars are then inserted at the lateral incisions. (**C**) The dissection is in the subplatysmal plane above the strap muscles. The boundaries of the subplatysmal working space are defined as follows: inferior border at the sternal notch, lateral borders at the edges of the sternocleidomastoid muscles, and superior border at the thyroid cartilage. Dissection of the thyroid lobe is then continued. (**D**) RLN is identified at the laryngeal insertion and dissected parallel to the trachea and downwards to the trachea.

TOETVA procedure have been previously described in both human and animal series^13,14^. Figure 2 details surgery. The cavity that was created had the subcutaneous tissue and platysma as the roof and the trachea, the sternohyoid and sternothyroid muscles on the floor. The muscles were then separated in the midline and the thyroid gland exposed. The gland was freed from the trachea (Fig. 2C). Hemostasis was then confirmed. The left RLNs were identified, exposed and monitored unilaterally on the same side of APS location (Fig. 2D). Direction of RLN dissection was from cranial to caudal (Fig. 2D).

#### CO_2_ insufflation

**Figure 3:**
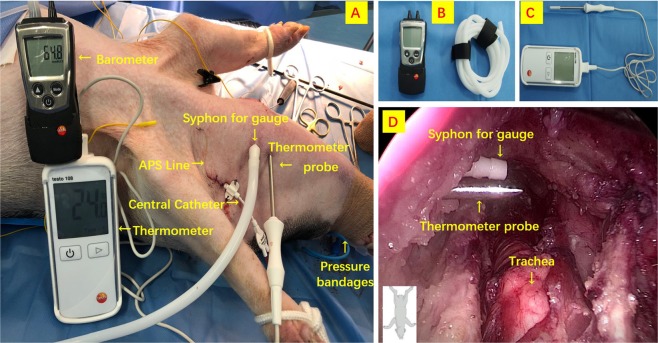
Airpocket temperature and pressure percutaneous determinations. (**A**) Pig model; (**B**) pressure gauge; (**C**) thermometer; (**D**) intra-neck endoscopic pictures of devices.

During dissection, CO_2_ insufflation was maintained at 6 mmHg with a flow rate of 15 L/min through the 10 mm central trocar. An elastic bandage was placed around the trocars vestibular surfaces to avoid air leakage (Figs 2b and 3a). The insufflator (Karl Storz-electronic endoflator #2643050, Tuttingen, Germany), was capable of regulating the gas flow and maintaining a constant positive pressure in the closed space.

#### Airpocket temperature and pressure determinations

Figure 3 describes procedures for airpocket temperature (Testo 108 Type T Thermometer, Lenzkirch, Germany) and pressure percutaneous determinations (Testo 510 Portable Pressure Indicator, Lenzkirch, Germany).

#### Protocol design

**Figure 4:**
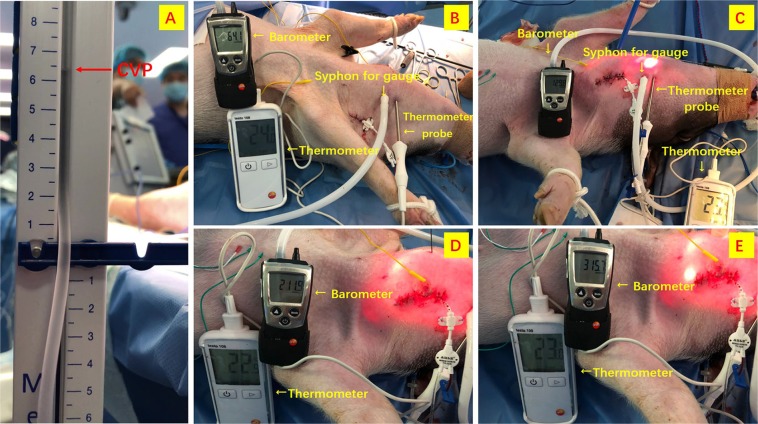
(**A**) Measurement of CVP. Twelve pigs were randomly divided into 4 groups according to CO_2_ insufflation pressure: (**B**) 1x CVP (Group I); (**C**) 2x CVP (Group II); (**D**) 3x CVP (Group III); (**E**) 5x CVP (Group IV).

After stabilization of blood gasses and hemodynamic parameters, baseline determinations were recorded before insufflation started. The animals underwent TOETVA. To minimize the potential bias of CO_2_ pressure on the contralateral nerves, solely unilateral (i.e. left) RLN dissection and monitoring was performed (same side of APS location). Twelve pigs (i.e. 12 left RLNs) were randomly divided into 4 groups (i.e. 3 animals, 3 left RLNs per group) according to CO_2_ insufflation pressure, which was maintained at a constant level for the duration of the experiment at one of the following levels: 1x CVP (Group 1), 2x CVP (Group 2), 3x CVP (Group 3), 5x CVP (Group 4) (Fig. 4). Carbon dioxide insufflation was maintained 180 minutes in all the pigs.

#### Main outcome measure

Recurrent laryngeal nerve and VN EMG parameter values (amplitude and latency) from ET electrodes were recorded continually. Testing was conducted without any manipulation or traction on the trachea to avoid changes to the contact area of the electrodes. Attempts were to keep the surgical field dry to avoid any artifacts and\or signal shunting and\or dispersion. Loss of signal was assumed in the event of signal failure or EMG signal <100 μV with a primary intact signal and adequate stimulation of 1–2 mA^11^. In case of LOS during the experimental procedures, the surgeon used the handheld stimulating probe for repeated tests of the RLN to observe any modification of amplitude or latency EMG signal and to elucidate location of injury. LOS was stratified according to Guidelines into segmental (type 1) or global injury (type 2)^11^.

#### Secondary outcomes measures

Oxygen saturation, heart rate, blood pressure, experimental time, blood gas (ABG) analysis (CO_2_ partial pressure, pH, O_2_ partial pressure), CVP, airpocket temperature and pressure were recorded. Blood analysis was performed using a analyzer (Radiometer ABL 90 FLEX Radiometer Medical, Bronshoj, Denmark). The samples were analyzed immediately in a laboratory next to the operating room.

#### Histopathology

Inferior laryngeal nerves segments were collected for histopathology to compare the morphological alterations caused by the experiments above. Hematoxylin and eosin (HE) histological staining will be used to distinguish and identify the RLN composition.

#### Statistical investigation

Values for each of the variables were obtained at base line, 10 minutes into the experimental procedure and after completion. All data are reported as mean ± standard deviation (SD). Statistical analyses were performed using the software package SPSS^®^ v. 22 for Windows^®^ (IBM, Armonk, New York, USA). Group comparisons were analysed with one-way analysis of variance. Group comparisons were performed using Student’s *t*-test. *P* < 0.05 was considered statistically significant. The sample size was estimated based on the principle of detecting a difference of −150 mcV between the mean of EMG amplitude with a 90% probability at p < 0.05, using power curve and sample size tools for 1-way analysis of variance (ie, a sample size of 24 nerves at risk - NAR - i.e. 12 animals) should provide 90% power to detect an amplitude difference of 150 mcV.

## Results

### Experimental model

APS and CVP placements, TOETVA were successfully performed in all cases with no occurrence of complications. Twelve left RLNs were finely exposed and monitored.

### Animals breads and baseline parameters

There were no significant difference between the Groups per age, weight, baseline parameters of blood pressure, heart rate, oxygen saturation, CVP, ABG and nerves EMG profiles (p > 0.05) (Table 1).

**Table 1:** Animal characteristics and baseline determinations.

	Group I	Group II	Group III	Group IV
Age ± SD (Days)	54.2 ± 2.3	55.1 ± 4.2	56.3 ± 2.2	55.8 ± 3.2
Gender (M/F)	3/0	3/0	3/0	3/0
Weight ± SD (Kg)	28.3 ± 1.6	28.9 ± 1.5	28.7 ± 1.3	28.5 ± 1.7
Experimental time (Min)	180 ± 0**	180 ± 0	83 ± 9.8	11.3 ± 2.1
**Blood pressure (mmHg)**				
systolic pressure	93.3 ± 8.5	96 ± 6.2	97.7 ± 9.9	95 ± 9.2
diastolic pressure	52.7 ± 6.1	49 ± 3.6	48.3 ± 7.6	53.3 ± 8.9
Heart rate (n/min)	69.7 ± 5.1	68.3 ± 9.3	62.7 ± 6.5	66.3 ± 6.7
Oxygen saturation (n/%)	97.7 ± 1.5	96.7 ± 3.1	96 ± 1	97.1 ± 2.1
CVP (cm H_**2**_O)	7.2 ± 0.7	6.7 ± 0.6	6.8 ± 0.4	7.1 ± 0.3
Cavity temperature (°C)	22.3 ± 0.6	23 ± 1	23.3 ± 0.6	22.7 ± 0.6
**ABG**				
pCO_2_ (mmHg)	22.4 ± 0.8	23.9 ± 1.1	24.2 ± 1.9	24.8 ± 1.7
pO_2_ (mmHg)	98.7 ± 1.2	99 ± 1	99.3 ± 0.6	99.7 ± 0.6
pH	7.52 ± 0.07	7.44 ± 0.07	7.46 ± 0.06	7.44 ± 0.14
**RLN**				
Amplitude(uv)	1046 ± 331.9	1253 ± 384.5	1067 ± 210.6	1015 ± 268.8
Latency(ms)	2.09 ± 0.35	1.98 ± 0.34	2.07 ± 0.19	2.12 ± 0.32
**VN**				
Amplitude(uv)	815.3 ± 217.5	859.3 ± 172.3	772.3 ± 70.2	785 ± 134.4
Latency(ms)	10.09 ± 0.19	9.79 ± 0.44	9.71 ± 0.19	9.75 ± 0.43

Group I: intraoperative pressure was kept at 1 times CVP; Group II: intraoperative pressure x2 CVP

Group III: intraoperative pressure x3 times CVP.; Group IV: intraoperative pressure x5 times CVP.

CVP: central venous pressure; ABG: arterial blood gas; RLN: recurrent laryngeal nerve; VN: vagus nerve.

*P < 0.05, **P < 0.01; analysis of the total four groups with Cochran’s and Mantel-Haenszel Chi-square (χ_CMH_^2^) test, while the variable of side was controlled.

### Hemodynamic parameters

**Figure 5:**
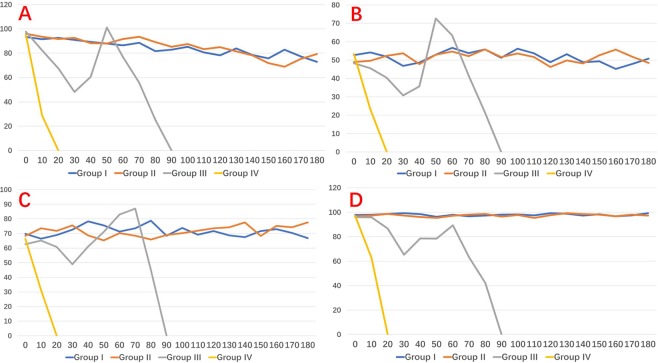
Hemodynamic parameters during experimental study, stratified per group. (**A**) Systolic pressure. (**B**) Diastolic pressure. (**C**) Heart rate. (**D**) Oxygen saturation.

There were no significant variation of systolic and diastolic pressure, heart rate and oxygen saturation in both Group I and II in respect to the baseline parameters (Fig. 5).

Group III measurements are presented in Fig. 5 and supplementary video (Video 2). Average experimental time was 83 ± 9.8 (72–91) minutes. In Group III, the hemodynamic indexes decreased and reached the lowest value parameter within 30 minutes. After rescue treatment (norepinephrine, dopamine, atropine intravenous injection), hemodynamic parameters gradually recovered. Yet, blood pressure continued to decline in the following 50 minutes; blood oxygen declined in 60 minutes; heart rate declined after 70 minutes mean (Fig. 5C). Average experimental time of Group IV was 11.3 ± 2.1^9–13^ minutes. In Group IV, hemodynamic parameters decrease rapidly, and no improvement was seen after rescue treatment (Fig. 5).

### Intraluminal temperature

**Figure 6:**
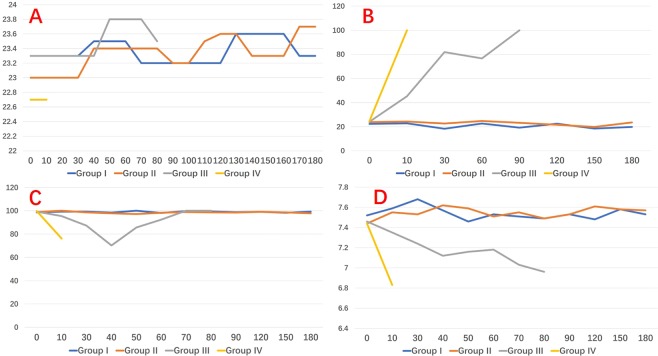
Figures for (**A**) intraluminal temperature, (**B**) CO_2_ partial pressure, (**C**) O_2_ partial pressure, and (**D**) pH.

There was no significant change in the intraluminal temperature between Groups. Temperature fluctuation within the groups was lesser than 1 °C (p > 0.05) (Fig. 6A).

### ABG analysis

Figure 6 display no significant variation for CO_2_ partial pressure, O_2_ partial pressure and pH value in Group I and II in respect to baseline parameters (Fig. 6B–D). In group III, CO_2_ partial pressure increased, decreased at 30 minutes after treatment (intravenous sodium bicarbonate), then-after incresed at 60 minutes (Fig. 6B). O_2_ partial pressure decreased reaching the lowest value after 40 minutes, returned to the normal level after treatment (high flow oxygen inhalation) (Fig. 6C). pH values had similar profiles (Fig. 6D). In Group IV, CO_2_ partial pressure increased consistently, O_2_ partial pressures and pH declined consistently. There was no change after rescue treatment (Fig. 6B–D).

### VN and RLN EMG profiles

**Figure 7:**
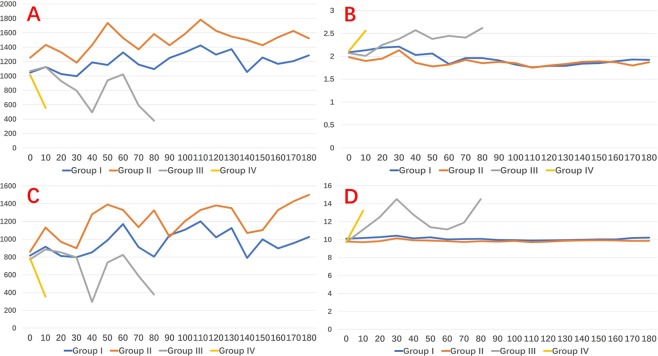
EMG signal profiles: (**A**) RLN amplitude; (**B**) RLN latency; (**C**) VN amplitude; (**D**) VN latency.

RLN and VN amplitude and latency profiles were stable during the Group I and II experiment all model (Fig. 7) The mean RLN and VN amplitude value at the end of experiment were respectively 1.287 ± 328.7 and 1.024.5 ± 237.6 Group I, 1.524 ± 353.2 and 1.499 ± 189.3 Group II. In group III, both VN and RLN amplitude decreased continuously, gradually increased at 40 minutes (during hemodynamic treatment), decreased again at 60 minutes until the end of the experiment (Fig. 7A,C). VN and RLN latency were gradually lengthened, shortened at 40 minutes, and recurring at 50 minutes (Fig. 7B,D and Video 2). In group IV, VN and RLN amplitude declined until the end of the experiment (Fig. 7A,C). VN and RLN latency lengthened (Fig. 7B,D). In nether Group III or IV, IONM couldn’t elucidate any precise location or segment of RLN injury (i.e. global injury, type 2). The mean RLN and VN amplitude value at the end of experiment were respectively 377 ± 105.5 and 378 ± 98.6 Group III, 553 ± 201.3 and 353 189.6 Group IV. No LOS was registered in the study groups.

### Histopathology

**Figure 8:**
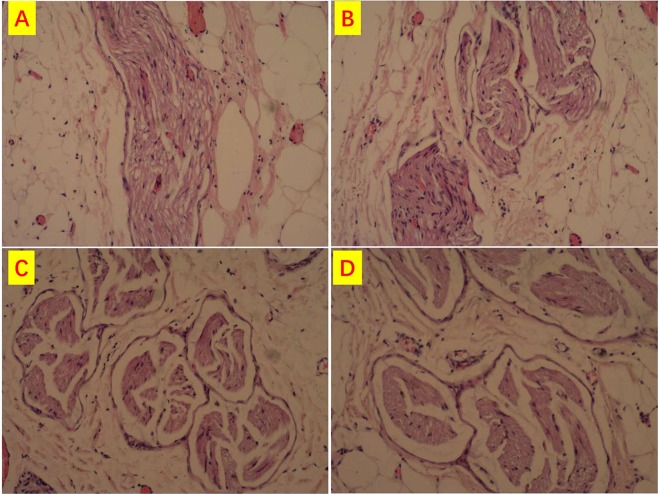
RLN consecutive longitudinal and transverse histological sections (HE x100). RLNs structure were preserved with normal axons, no fibrosis, and no vacuolization and without loss of myelinated fibres. (**A**) Group I, (**B**) Group II, (**C**) Group III, (**D**) Group IV.

Consecutive longitudinal and transverse histological sections with HE stain, at high magnification (HE x100), showed that RLNs structure were preserved without loss of myelinated fibres (Fig. 8). Rappresentative sections are depicted in Fig. 8. Figure 8 shows normal axons, no fibrosis, and no vacuolization. Nerves structure were identical to the contralateral RLN (Fig. 8).

### Time period

**Figure 9:**
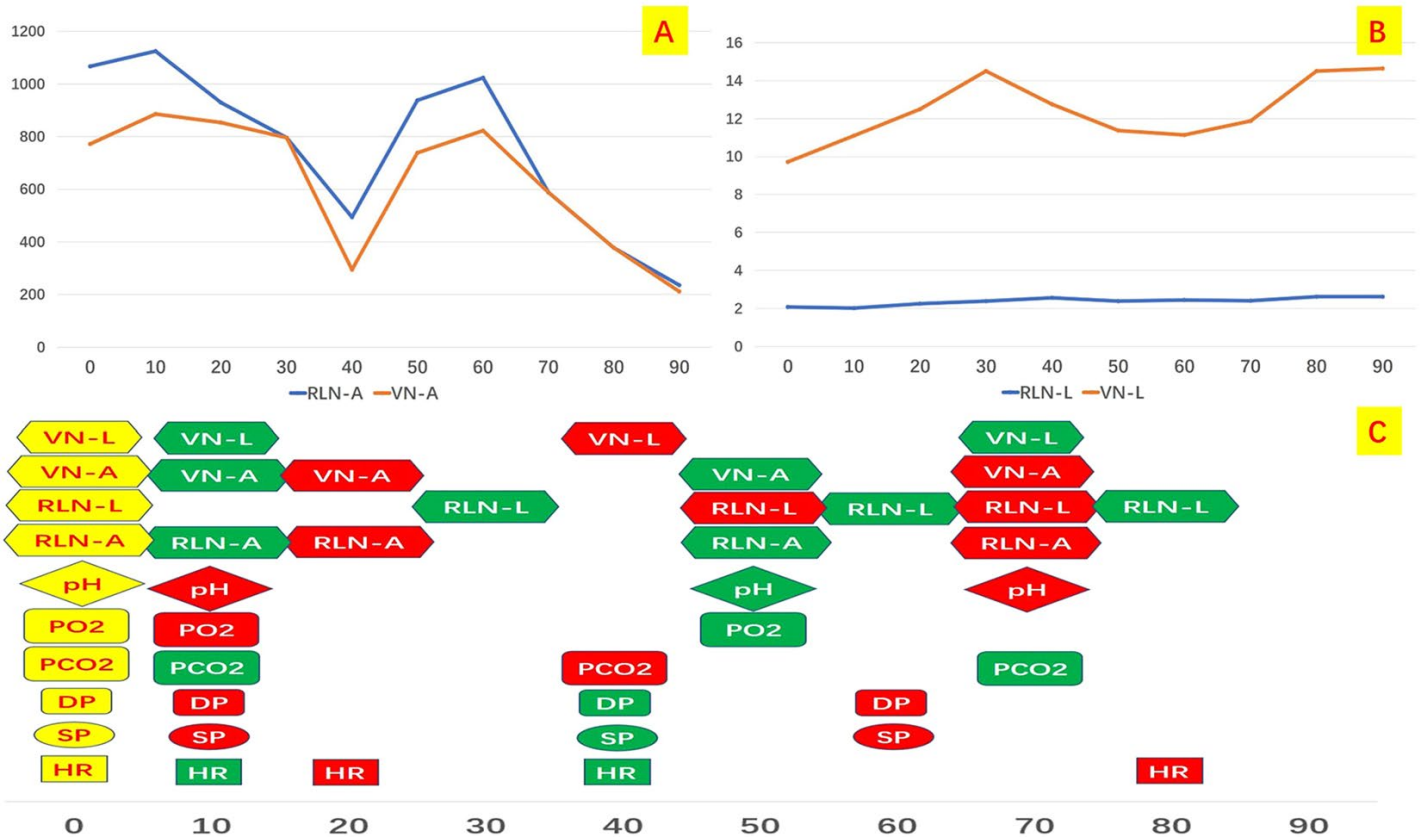
Group III EMG profiles during experiment. Time trends for (**A**) amplitude values; (**B**) latency; (**C**) metabolic, hemodynamic and EMG parameters variations. Green dots: increase values. Red dots: decrease values. VN: vagal nerve. RLN: recurrent laryngeal nerve. L: latency. A: amplitude. PO_2_: PO_2_ partial pressure. PCO_2_: CO_2_ partial pressure. DP: diastolic pressure. SP: systolic pressure. HR: heart rate.

**Figure 10:**
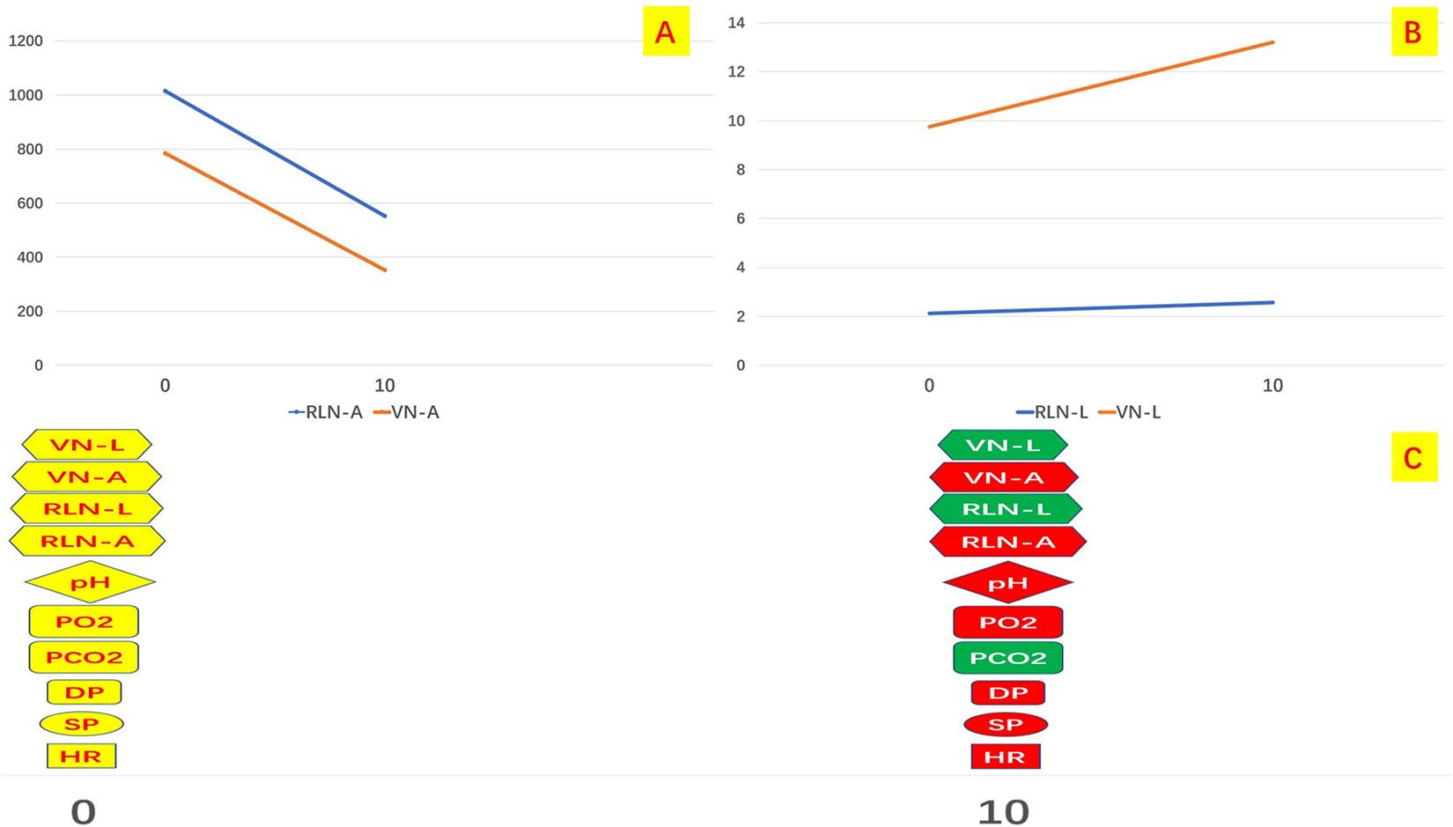
Group IV EMG profiles during experiment. Time trends for (**A**) amplitude values; (**B**) latency; (**C**) metabolic, hemodynamic and EMG parameters variations. Green dots: increase values. Red dots: decrease values. VN: vagal nerve. RLN: recurrent laryngeal nerve. L: latency. A: amplitude. PO_2_: PO_2_ partial pressure. PCO_2_: CO_2_ partial pressure. DP: diastolic pressure. SP: systolic pressure. HR: heart rate.

Figures 9 and 10 depict time trends analysis for metabolic, hemodynamic and EMG values variations in Group III and IV. Interestingly, EMG profiles variations were not anticipated by nether hemodynamic and metabolic parameters.

## Discussion

TOETVA has added a new option to the surgical treatment of thyroid disease. CO_2_ insufflation is required during TOETVA similar to other endocrine endoscopic techniques.

Additional CO_2_ insufflation may be necessary for adequate exposure of intra-neck structures.

The lack of studies that address the safety of CO_2_ neck insufflation on RLN function stimulated us to investigate the effect of different levels of insufflation pressure on nerve EMG profiles (amplitude and latency), and metabolic and hemodynamic parameters in a TOETVA animal prospective randomized model.

We hypothesized that differences in CO_2_ insufflation pressure could result in different RLN function effects.

The idea that high CO_2_ insufflation pressure may affect the RLN function is new. This is the first animal model to analyze gas insulation on the RLN.

Under continuous intraoperative RLN monitoring and after histopathology evaluation, carbon dioxide neck insufflation appears to be safe at 6 mmHg for the RLN. RLNs thresholds were remarkably constant in experimental Groups I and II with long exposure times. Metabolic and hemodynamic parameters in Group I and II TOETVA animal model were also within the normal range.

In both Group III and IV, both VN and RLN function were impaired.

The results of this study cannot definitively exclude the possibility that CO_2_ at high pressures could damage the RLN. Higher CO_2_ insufflation pressures determined combined event of EMG amplitude decrease and latency increase^11,12^. According to Schneider *et al*., the important combined EMG event is commonly indicative of impending RLN injury^12^.

Additionally, the relationship between CO_2_ concentration and magnitude of EMG signal could be explained by second hypotesis. Hence, EMG is a complicated signal, which is controlled also by the central nervous system^15^. Other possible causative factors of the combined RLN EMG event are the direct intrinsic affect on signals from biochemical factors (presumably reflecting O_2_ partial pressures and pH decline, CO_2_ partial pressure increase) and reduced blood flow^15^.

Furthermore, IONM is an adjunct technique for localizing the site of RLN injury^11^. In the present study, IONM couldn’t locate any precise segment of RLN injury presumably defining a type 2 global nerve injury^11,12^.

No LOS was registered in any Groups. In detail, the mean RLN and VN amplitude value at the end of experiment were: 1287 ± 328.7 and 1024.5 ± 237.6 Group I, 1524 ± 353.2 and 1499 ± 189.3 group II, 377 ± 105.5 and 378 ± 98.6 Group III, 553 ± 201.3 and 353 ± 189.6 Group IV. Histological sections of Group III and IV RLNs revealed structure preservation, without loss of myelinated fibres, normal axons, no fibrosis, and no vacuolization.

Even though we found that the RLN fuction was altered in the CO_2_ insufflation pressure more than three times than that of the CVP, it may be affected by the systemic alteration of the hemodnamic status of the pigs such as the hypotension, bradycardia, hypercarbia and acidosis, which authors identified and described in the result section. Interestingly, EMG profiles variations were not anticipated by nether hemodynamic and metabolic parameters (Figs 9 and 10).

The study was designed to insufflate the CO_2_ at the maximal pressure of two, three, and even five times than that of the CVP, which will never happen in the endoscopic thyroid surgery in human. In transoral endoscopic thyroidectomy which is becoming popular nowadays, surgeons usually insufflate the CO_2_ at the maximal pressure of 6 mmHg which may be equal or less than that of the patient’s central vein. It is essential to maintain the CO_2_ insufflation pressure as low as possible not only to avoid systemic hazards including hypotension, bradycardia, hypercarbia and acidosis but to avoid local complication such ac subcutaneous gaseous emphysema which may develop if the insufflation pressure reaches over 10 mmHg. In addition, it may be beneficial to maintain the low CO_2_ insufflation pressure to prevent the possible CO_2_ pulmonary embolism which was recently reported in the literature.

Our results confirm previous report by Bellantone *et al*., that eccessive dioxide insufflation in the neck cause adverse effects on hemodynamic and blood gas levels^16^. Hemodynamic parameters were significantly altered in both Group III and IV in respect to the baseline parameters. The use of higher insufflation pressures should be avoided due to the potential risks^16^. Close monitoring of metabolic and hemodynamic parameters are recommended during TOETVA, especially if additional CO_2_ insufflation is necessary for better exposure, as they may help an early detection of any abnormality.

The anatomy of the neck and the chest of pig is quite different from human. As the consequence, the amount of insufflated gas and its impact on the cardiovascular system (and on the nervous system including RLN) in pigs will be quite different from those in humans. This may be important limitation of the study.

In conclusion, it’s dangerous for the surgeon to increase CO_2_ beyond 6 mm Hg insufflation.

## Supplementary information

measurement of the central venous pressure

The relationship between the changes of vital signs and amplitude and latency of VN,RLN in Group III

## Data Availability

The datasets used and/or analysed during the current study are available from the corresponding author on reasonable request.

## References

[1] Russell JO (2018). Transoral Thyroid and Parathyroid Surgery Vestibular Approach: A Framework for Assessment and Safe Exploration. Thyroid..

[2] Dionigi G, Chai YJ, Tufano RP, Anuwong A, Kim HY (2018). Transoral endoscopic thyroidectomy via a vestibular approach: why and how?. Endocrine..

[3] Anuwong A (2018). Transoral endoscopic thyroidectomy vestibular approach (TOETVA): indications, techniques and results. Surg Endosc..

[4] Russell JO (2017). Transoral thyroidectomy and parathyroidectomy - A North American series of robotic and endoscopic transoral approaches to the central neck. Oral Oncol..

[5] Choi YM (2017). Postoperative analgesic efficacy of single-shot and continuous transversus abdominis plane block after laparoscopic cholecystectomy: A randomized controlled clinical trial. J Clin Anesth..

[6] Niknam F, Saxena A, Niles N, Budak UU, Mekisic A (2014). Does irrigation of the subdiaphragmatic region with ropivacaine reduce the incidence of right shoulder tip pain after laparoscopic cholecystectomy? A prospective randomized, double-blind, controlled study. Am Surg..

[7] Cha SM (2012). Peritrocal and intraperitoneal ropivacaine for laparoscopic cholecystectomy: a prospective, randomized, double-blind controlled trial. J Surg Res..

[8] Rice ASC (2013). Transparency in the reporting of *in vivo* pre-clinical pain research: The relevance and implications of the ARRIVE (Animal Research: Reporting *In Vivo* Experiments) guidelines. Scand J Pain..

[9] Zhao Y (2019). Experimental study of needle recording electrodes placed on the thyroid cartilage for neuromonitoring during thyroid surgery. Br J Surg..

[10] Liu X (2018). Laryngeal nerve morbidity in 1.273 central node dissections for thyroid cancer. Surg Oncol..

[11] Randolph GW (2011). Electrophysiologic recurrent laryngeal nerve monitoring during thyroid and parathyroid surgery: international standards guideline statement. Laryngoscope..

[12] Schneider R (2019). Prediction of Postoperative Vocal Fold Function After Intraoperative Recovery of Loss of Signal. Laryngoscope..

[13] Zhang, D. *et al*. Feasibility of Continuous Intraoperative Neural Monitoring During Transoral Endoscopic Thyroidectomy Vestibular Approach in a Porcine Model. *J Laparoendosc Adv Surg Tech A*, 10.1089/lap.2018.0054 (2018). 10.1089/lap.2018.0054 29746219

[14] Sun H, Dionigi G (2019). Applicability of transoral robotic thyroidectomy: Is it the final solution?. J Surg Oncol..

[15] Reaz MB, Hussain MS, Mohd-Yasin F (2006). Techniques of EMG signal analysis: detection, processing, classification and applications (Correction). Biol Proced Online..

[16] Bellantone R (2001). Arterial PCO_2_ and cardiovascular function during endoscopic neck surgery with carbon dioxide insufflation. Arch Surg..

